# Differential Diagnosis of Fulminant Myocarditis and Acute Coronary Syndromes in the Case of Failure of Coronary Angiography: A Case Report

**DOI:** 10.3389/fcvm.2021.690974

**Published:** 2021-11-30

**Authors:** Xiangfei Huang, Yi Gao, Fuzhou Hua, Jun Ying

**Affiliations:** ^1^Department of Anesthesiology, The Second Affiliated Hospital of Nanchang University, Key Laboratory of Anesthesiology of Jiangxi Province, Nanchang, China; ^2^Department of Critical Care Medicine, The Second Affiliated Hospital of Nanchang University, Nanchang, China

**Keywords:** fulminant myocarditis, acute coronary syndromes, coronary artery anomaly, coronary angiography, extracorporeal membrane oxygenation, intra-aortic balloon pump

## Abstract

Fulminant myocarditis (FM) is a severe disease with a rapidly progressive and life-threatening course caused mainly by viral infection. The symptoms, laboratory findings, and presence of ECG changes resemble acute coronary syndrome. Therefore, coronary angiography is usually helpful in making the appropriate diagnosis. However, failure to obtain complete coronary artery images due to coronary artery anatomic variations poses a challenge for the diagnosis of FM. Here, we report a case of FM preliminarily diagnosed as acute coronary syndrome (ACS) due to the presence of coronary artery anomaly.

## Introduction

Fulminant myocarditis (FM) is a severe disease with a rapidly progressive and life-threatening course. Viral infection-induced inflammation of the heart muscle is the most common cause (1). The symptoms of FM are non-specific, such as dyspnea, chest pain, and arrhythmia. FM can lead to cardiogenic shock, and severe cases can lead to multiorgan failure immediately after onset. These presenting symptoms, as well as electrocardiogram changes and laboratory findings, such as elevated troponin I and creatine kinase-MB (CK-MB) (2), can be found in both FM and acute coronary syndrome (ACS), making the diagnosis difficult. Coronary angiography, which provides information on whether an acute blockage is occurring within the coronary artery system, is critical to distinguishing FM from ACS (3). However, in the case of coronary artery variation, coronary angiography may be unable to provide a complete assessment of the coronary arteries, making the diagnosis of FM difficult. Here, we report a case of FM with coronary artery anomaly and present the diagnosis and treatment process.

## Case Presentation

**Figure 1:**
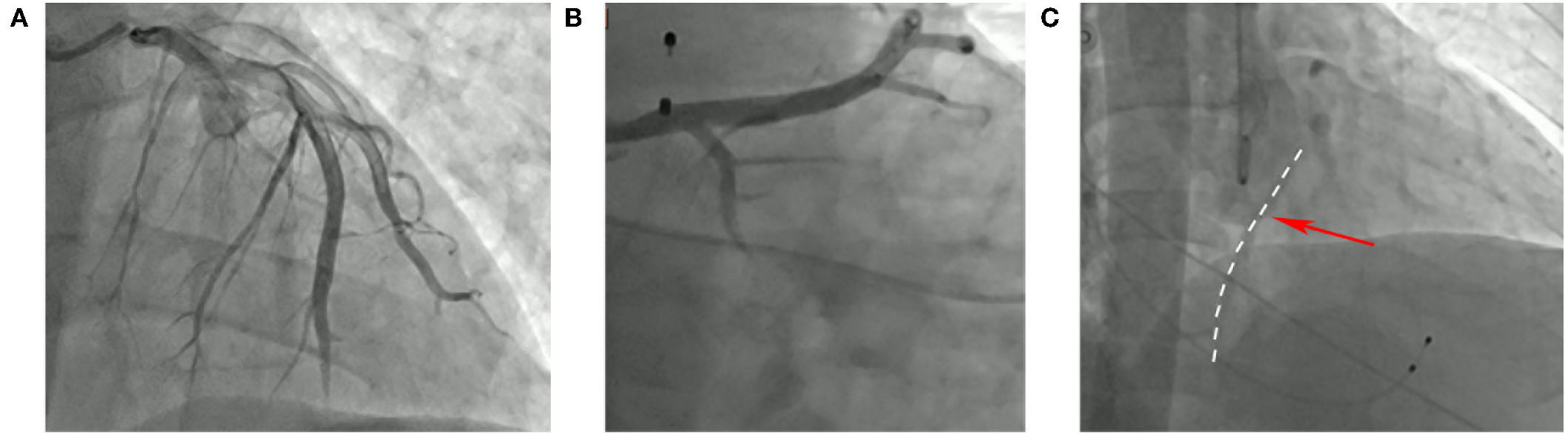
Angiographic image of this patient. **(A)** (AP + CRA30°) the left anterior descending branch. **(B)** (RAO30° + CAU20°) the circumflex branch. **(C)** (AP + CRA20°) the right coronary artery.

A 34-year-old gentleman suddenly developed colic-like, persistent anterior chest pain. Fifty minutes later, he was sent to the emergency room of the local hospital. The emergency ECG showed “Sinus arrest and junctional rhythm; ST-elevation in leads II, III, and avF, and ST-depression in leads I, avL, and V1–V6” (see Supplementary Materials). Biochemical examination showed “hypersensitive troponin I (TnI): 27.052 ng/ml (normal range: 0–0.78), myoglobin (MB): 4,102 ng/ml (normal range: 0-85), creatine kinase-MB (CK-MB): 297 U/L (normal range: 0–24).” Given the high probability of acute myocardial infarction, coronary angiography was immediately performed but showed no signs of blockage or stenosis in the left main, left anterior descending branch (Figure 1A), or left circumflex coronary artery (Figure 1B). However, the ostium of the right coronary artery was unable to be engaged with the angiographic catheter due to an abnormal origin between the non-coronary sinus and the left coronary sinus (“pigtail” catheter angiography, Figure 1C). Ventricular fibrillation occurred many times during the operation, and repeated electrical cardioversion and defibrillation were performed. Amiodarone and esmolol were used to control ventricular rate and noradrenaline was used to maintain blood pressure. To avoid circulatory collapse, intra-aortic balloon pump (IABP), endotracheal intubation and ventilator-assisted ventilation, and vein-artery extracorporeal membrane oxygenation (ECMO) were applied. Subsequently, he was transferred to the cardiovascular care unit of our hospital for further treatment.

Cardiologists thought that this patient was young, with no known risk factors for coronary artery disease, such as hypertension, hyperlipidemia, smoking, or family history, and so the diagnosis of acute myocardial infarction may be incorrect. After carefully reviewing the coronary angiography images, they found that the left coronary artery was the dominant feeding artery, and that the right coronary artery was able to be observed (Figure 1C). Meanwhile, Color Doppler echocardiography showed that the left ventricular systolic function was decreased, and the ejection fraction (EF) was only 26%, which is inconsistent with the characteristics of inferior wall myocardial infarction. Additionally, laboratory findings revealed coxsackie B IgM (+), which is important supporting evidence of acute myocarditis (4). Combined with the rapidly progressive clinical presentation, laboratory findings, ECG changes (see Supplementary Materials), and Doppler echocardiography, the patient met diagnostic criteria for FM and appropriate treatment was initiated (5).

The patient then developed multi-organ dysfunction, with associated circulatory, respiratory, renal, and hepatic impairment. Along with methylprednisolone therapy, bedside continuous renal replacement therapy (CRRT) was used. IABP and ECMO were used for a total of 6 days. After 10 days, once the oxygen saturation was stable and sinus rhythm was recovered, the endotracheal tube and temporary pacemaker were removed. CRRT was intermittently used for a total of 12 days. The use of methylprednisolone was gradually reduced and completely stopped after 25 days of use. Once the patient was hemodynamically stable, metoprolol tartrate was initiated to prevent ventricular remodeling. The patient and his family agreed and actively cooperated with the above treatment. The tests and examinations showed that the condition of the patient had improved (Table 1), and he was discharged after 27 days in hospital.

**Table 1:** Timeline with the tests and examinations.

**DATE**	**EF (%) (50–70)**	**PC T(ng/mL) (0–0.5)**	**CRP(mg/L) (0–8)**	**BNP (pg/mL) (0–100)**	**TnI (ng/mL) (0–0.78)**	**MB (ng/mL) (0–85)**	**CK-MB(U/L) (0–24)**
1105	26	15.01	129	–	–	8,688	773.6
1106	–	–	–	–	–	6,575.4	267.9
1107	–	–	182	134.8	–	9,884.2	143.6
1108	33	–	–	331.53	–	1,0261	93.2
1109	55	2.73	–	306.1	–	–	–
1110	57	–	91	–	–	–	–
1111	50	–	–	485.18	–	1,853.3	33.9
1112	56	31.63	–	–	–	1,468.7	33.3
1113	56	–	–	–	–	–	36.7
1114	–	–	–	508.62	15.96	–	–
1115	–	5.89	73.7	–	–	–	–
1116	–	–	–	910.88	21.79	–	40.1
1117	–	–	–	–	–	–	–
1118	–	2.62	90.6	–	–	5,008.8	35.9
1119	59	–	–	816.86	8.08	–	–
1120	–	–	–	–	–	1,787.9	57.5
1121	–	–	–	571.84	3.76	4,432	40.6
1122	–	–	–	–	–	3,193.1	23.6
1123	–	0.63	–	–	–	4,007.7	31
1124	–	–	–	531.61	1.05	–	–
1125	–	–	–	–	–	3,244.2	26.4
1128	57	–	27.5	435.62	1.03	–	31.8
1201	–	–	–	–	–	–	29.9

## Discussion

As a special form of acute myocarditis, FM is much more severe and can cause rapid heart failure, fatal hemodynamic compromise, severe hypotension, and cardiogenic shock. Diddle et al. (6) reported that the overall survival rate is only 61%. The clinical presentation of FM is indistinguishable from ACS in patients, especially those with the onset of acute chest pain and arrhythmia. The ECG changes and laboratory findings like TnI and ck-MB are also non-specific. The preexisting symptom of viral infection may be helpful in distinguishing FM from ACS, but can also be absent. Thus, coronary angiography is recommended as an essential differential diagnosis tool in the early course of the disease (3). However, in rare cases, as we described, coronary angiography cannot provide complete information due to anatomic variations of the coronary arteries. This particular situation makes distinguishing between FM and ACS challenging.

About 1% of adults have coronary artery malformations, most of which are anomalies of origin and distribution (7). The abnormal origin of coronary arteries may cause the failure of angiography and result in misdiagnosis and mismanagement. Cross-sectional imaging, like computed tomography (CT), is the preferred approach to evaluate anatomic variation of the coronary arteries (8). In addition, as a non-invasive examination, cardiac magnetic resonance (CMR) avoids radiation and contrast agents and has higher accuracy (92%) and sensitivity (88%) than x-ray coronary angiography in identifying the origin of the proximal coronary arteries (9).

Meanwhile, CMR also plays a vital role in the assessment of myocarditis. Abdel-Aty et al. reported that CMR could provide an 85% diagnostic accuracy for myocarditis when patients meet two of the three sequences (10). Known as the gold standard for the diagnosis of FM, endomyocardial biopsy (EMB) is reasonable when patients present with new-onset heart failure within 2 weeks and unexplainable hemodynamic compromise with or without a dilated left ventricle (11).

However, the application of these auxiliary examinations is sometimes limited in clinical practice. In this case, the condition of the patient was critical and necessitated life-support treatments, so the examinations mentioned above, such as CT, MRI, and EMB, could not be obtained. Under these circumstances, we carefully reviewed the detailed history, symptoms, physical signs, laboratory findings, examinations, and disease course. The diagnosis was corrected in time, and the patient received timely treatments.

Fulminant myocarditis (FM) is a potentially fatal disease with a rapid onset and progressive course. However, long-term prognosis is superior to that of acute myocarditis if the patients live through the acute disease phase (12). Although there are no criteria for the treatment of FM, Li et al. (13) reported that early combination of life-support treatments, like ECMO and IABP, antiviral drugs, intravenous immunoglobulin (IVIG) administrations, and glucocorticoid treatments significantly decrease in-hospital mortality. Long-term pressor drugs like noradrenaline and dopamine without life-support treatment accelerate mortality (13).

## Conclusion

Fulminant myocarditis (FM) is a potentially fatal disease that is hard to distinguish from ACS. In rare cases, as we described, anatomic variations of the coronary arteries will make the diagnosis of FM more difficult. Thus, comprehensive assessments should be considered, including a detailed history, symptoms, physical signs, laboratory findings, auxiliary examinations, and disease course. A better long-term prognosis can be achieved when patients are treated with life-support treatments, such as ECMO and IABP, in the early period.

## Data Availability Statement

The original contributions presented in the study are included in the article/Supplementary Material, further inquiries can be directed to the corresponding author.

## Ethics Statement

The studies involving human participants were reviewed and approved by the Ethics Committee of the Second Affiliated Hospital of Nanchang University. The patients/participants provided their written informed consent to participate in this study. Written informed consent was obtained from the individual(s) for the publication of any potentially identifiable images or data included in this article.

## Author Contributions

XH and YG wrote this paper. YG, JY, and FH managed the patient. JY and FH revised this paper. All authors contributed to the article and approved the submitted version.

## Conflict of Interest

The authors declare that the research was conducted in the absence of any commercial or financial relationships that could be construed as a potential conflict of interest.

## Publisher's Note

All claims expressed in this article are solely those of the authors and do not necessarily represent those of their affiliated organizations, or those of the publisher, the editors and the reviewers. Any product that may be evaluated in this article, or claim that may be made by its manufacturer, is not guaranteed or endorsed by the publisher.

## Supplementary Material

Supplement S1 ECG on November 3, 2020 (50 min after onset).

Supplement S2 ECG on November 19, 2020.

Supplement S3 ECG on December 18, 2020.
